# Predictors of Change in Functional Outcome at six months and twelve months after Severe Injury: A Retrospective Cohort Study

**DOI:** 10.1186/s13017-018-0217-y

**Published:** 2018-12-03

**Authors:** Aidan Lyanzhiang Tan, Yi Chiong, Nivedita Nadkarni, Jolene Yu Xuan Cheng, Ming Terk Chiu, Ting Hway Wong

**Affiliations:** 1 Preventive Medicine, National University Hospital, Singapore, Singapore; 2 Health Services Research Unit, Singapore General Hospital, Singapore, Singapore; 3 Department of Rehabilitation Medicine, Singapore General Hospital, Singapore, Singapore; 4 Centre for Quantitative Medicine, Duke-NUS Graduate Medical School, Singapore, Singapore; 5 National Trauma Unit, Tan Tock Seng Hospital, Singapore, Singapore; 6 Department of General Surgery, Tan Tock Seng Hospital, Singapore, Singapore; 7 General and Trauma Surgery, Singapore General Hospital, Singapore, Singapore; 8 Health Services and Systems Research, Duke-NUS Graduate Medical School, Singapore, Singapore

**Keywords:** Trauma, Quality of life, Functional outcomes, Severe injury, Singapore, Asia

## Abstract

**Background:**

There is increasing focus on long-term survival, function and quality-of-life for trauma patients. There are few studies tracking longitudinal changes in functional outcome over time. The goal of our study was to compare the Glasgow Outcome Scale-Extended (GOSE) at 6 months and 12 months in blunt trauma survivors with an Injury Severity Score (ISS) of more than 15.

**Methods:**

Using the Singapore National Trauma Registry 2011–2013, patients with 6-month GOSE and 12-month GOSE scores were analysed. Patients were grouped into three categories—those with the same score at 6 months and 12 months, an improvement in score, and a worse score at 12 months. Ordinal regression was used to identify risk factors for improved score. Patients with missing scores at either 6 months or 12 months were excluded.

**Results:**

We identified 478 patients: 174 had an improvement in score, 233 stayed the same, and 71 had worse scores at 12 months compared to 6 months. On univariate ordinal regression, the following variables were associated with same or better function at 12-months compared to 6-months: male gender, being employed pre-injury, thoracic Abbreviated Injury Scale (AIS) of 3 or more, anatomical polytrauma (AIS of 3 or more in 2 or more body regions), and road traffic injury mechanism. Older age, low fall, increasing Charlson comorbidity scores, new injury severity score, and head and neck AIS of 3 or more were associated with worse function at 12 months compared to 6 months. ISS and revised trauma score were not significant predictors on univariate or multivariable analysis.

On multivariable ordinal regression, motor vehicle mechanism (OR 2.78, 1.51–5.12, *p* = 0.001) was associated with improved function, while male gender (OR 1.36, 95% CI 1.02–1.82, *p* = 0.039) predicted improved function at 12 months.

**Conclusions:**

Females experience worse functional outcomes at 12 months, potentially due to majority of female injuries being low falls in the elderly. In contrast, motor vehicle injury patients had better functional outcomes at 12 months. Additional interventional strategies for high-risk groups should be explored.

## Introduction

Trauma is a cause of chronic morbidity and disability [1]. Trauma survivors may suffer from activity limitations, reduced or inability to work in addition to other participation restrictions [1–4]. Socio-economic consequences are often present [3, 5, 6]. There are several scales that are available to measure quality of life in trauma survivors but few studies have tracked longitudinal changes in functional outcome over time. Glasgow Outcome Scale-Extended (GOSE) is a measure of overall disability, originally used to assess global outcome in traumatic brain injury survivors, and also used for overall outcomes for trauma survivors [7–12]. Recovery trajectories may differ based on patient characteristics and other factors [10, 12–23]. Outcomes have also been known to vary depending on healthcare system [22, 24–27]. Routine registry data can help provide a good reflection of trauma outcomes [8, 11, 23, 28–30].

In Singapore, trauma is the leading cause for hospitalisation, and one of the major causes of mortality [31]. Using the registry information from the Singapore National Trauma Registry, established in 2011, we aimed to evaluate factors associated with improvement in function, measured by change in GOSE score at 12 months post-trauma, for blunt trauma survivors with severe injuries. The secondary aim was to compare the characteristics of responders to non-responders, given that our registry GOSE scores are generally obtained during office hours.

## Methods

### Data source and data collection

A retrospective multi-centre cohort study was conducted using the Singapore National Trauma Registry data. It covered all patients admitted via the emergency department in all public-sector hospitals in Singapore from the year 2011 to 2013, regardless of nationality. Details of the registry data collection and processing have been described [32, 33].

GOSE scoring was conducted using the standardised structured interview via telephone call during office hours by registry officials unrelated to the study as part of the routine registry data collection process. This included indirect methods to interview patient caregivers, as direct interview with all patients was impracticable [13, 34]. The GOSE has been validated for administration via proxy or direct interview with the patient [35], with no difference when administered via telephone or otherwise [36, 37].

GOSE scoring was either performed face-to-face (if patients were still warded at the acute hospital at the time), or by phone. Consent for the GOSE scoring was obtained prior to proceeding with the scoring questions. The phone numbers were obtained from hospital records, which contain both home and cell phone numbers. In the event that the telephone call did not get through, another attempt was done at a separate time and day, for up to five separate occasions before a “missing” entry made.

### Study design

Retrospective data from January 2011 to December 2013 was extracted. The association between change in GOSE scores and patient, injury, and clinical factors was examined.

### Study population

We included adult patients (age 18 years and older) who sustained blunt injuries based on International Classification of Diseases, ninth revision, diagnostic codes 800–959.9, excluding 905–909.9. As the Singapore NTR only contains GOSE outcome data for patients with at least injury severity score (ISS) of 16, no data was available for those with ISS < 16 [38]. Patients with missing GOSE scores at either timepoint for the primary analysis were also excluded.

### Outcome measures

The primary outcome measure was change in function at 12 months, compared to 6 months post-trauma. We measured and compared the GOSE score at 6 months and 12 months. These were categorised as similar (no change in scores), improved (increased), or worse (decreased) score at 12 months relative to the 6 month score.

### Covariates

We extracted patient demographics and injury details (mechanism and severity) from the trauma registry.

Patient demographics were age, gender, and pre-injury employment status. Pre-injury employment status was a binary variable defined as “employed” or “unemployed”. Patient comorbidity was measured using the Charlson comorbidity index (CCI) [39].

Injury mechanisms were coded in the registry as motor vehicle accident, fall, interpersonal violence, machinery, tools/objects, sports, unknown and others. We re-categorised injury mechanisms as ‘low fall’ (equivalent to falls from standing, chair or bed), [33, 40] ‘high fall’ (falls from heights greater than 0.5 m) [33, 40], ‘road traffic’ (motor vehicle accident, pedestrian, and motorcycle injuries), and ‘others’.

We measured injury severity using the ISS, New Injury Severity Score (NISS) [26], Revised Trauma Score (RTS) [41]. The pattern of injury was described using an Abbreviated Injury Scale (AIS) score of 3 or greater at each AIS region [42].

### Statistical testing

Patient characteristics at baseline were summarised by mean (standard deviation), median (inter-quartile range), or frequency (%) as appropriate. Univariate comparisons between change in GOSE scores and predictors of interest was conducted using two-sample *t* tests or Mann-Whitney tests as appropriate, and chi-square tests, depending on variable type.

A multivariable ordinal regression model was fit to analyse patient group differences between those with similar/improved GOSE scores, and those with worse scores. Variables identified as having statistically significant associations (*p* <  0.05) in the univariate regression were included. Variables not statistically significant but deemed clinically meaningful were also included.

For the secondary analysis, we compared patient group differences between those without GOSE scores (non-responders to the registry calls), and those with GOSE scores (responders to registry calls). Univariate analysis using two-sample *t* tests or chi-square tests was conducted, depending on variable type.

STATA v.13 was used.

### Ethical issues

Study and data collection protocols and processes were reviewed and approved by the respective hospital institutional review boards.

## Results

### Descriptive analysis

We identified 3013 potentially eligible patients from the registry and excluded 2072 as having no GOSE scores at all/non-responders to the telephone calls. Of the remaining 941 patients meeting the inclusion criteria, we further excluded 18 (1.9%) with missing 6-month GOSE scores and 445 (47.3%) with missing 12-month GOSE scores. Only 478 (50.8%) had complete GOSE scores at both 6-month and 12-month timepoints.

Of the 478 patients, compared to their 6-month score, one third had improved GOSE scores (*n* = 174, 36.4%), and 71 (14.9%) had worse GOSE scores at 12 months. The remainder (*n* = 233, 48.7%) had similar scores.

The mean age was 61.1 years, with males accounting for over two-thirds of the sample (*n* = 331, 69.2%). Majority were Singapore citizens or permanent residents (*n* = 418, 87.4%). Less than half were employed prior to their injury event (*n* = 225, 47.1%). The most common mechanism of injury was from low falls (*n* = 214, 44.8%). Majority had sustained head and neck injuries (*n* = 363, 75.9%). Summary statistics are presented in Table 1.

**Table 1:** Summary statistics

		Number (%)/median (IQR)/mean (SD)	
Demographics	Age	61.1	(19.9)
	Males	331	(69.2%)
Injury scores	Injury Severity Score (ISS)	22	(17, 26)
	New Injury Severity Score (NISS)	29	(22, 38)
	Revised Trauma Score (RTS)	7.84	(3.80, 7.84)
Charlson comorbidity score	0	339	(70.9%)
	1	93	(19.5%)
	2	31	(6.5%)
	> 2	15	(3.1%)
Citizenship	Singapore citizen/permanent resident	418	(87.4%)
Pre-injury employment status	Employed	225	(47.1%)
Mechanism of injury	Low fall	214	(44.8%)
	High fall	77	(16.1%)
	Pedestrian	13	(2.7%)
	Cyclist	13	(2.7%)
	Motorcycle	63	(13.2%)
	Motor vehicle	22	(4.6%)
Injury Region	Head and neck	363	(75.9%)
	Face	8	(1.7%)
	Chest	126	(26.4%)
	Abdomen and pelvic contents	48	(10.0%)
	Extremities and pelvic girdle	95	(19.9%)
	Polytrauma	135	(28.2%)

### Univariate analysis

**Fig. 1:**
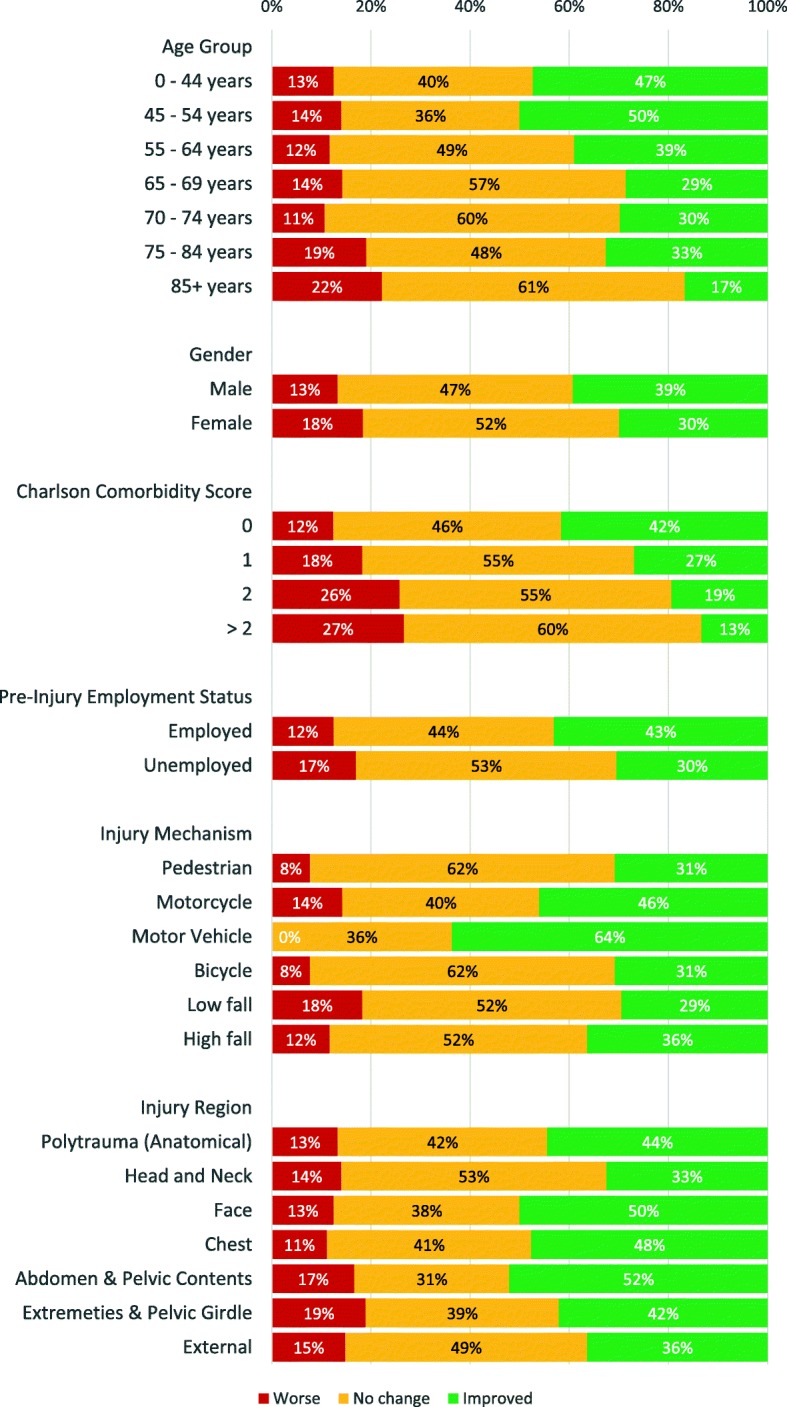
Distribution of GOSE score change according to patient and clinical factors

Functional outcomes at 12 months compared to 6 months were the same or better for male patients, or those who were employed prior to injury. Certain injuries and patterns were more likely to be associated with improved or similar functional outcomes: motor vehicle mechanism, those with injuries on the chest (AIS 3 or more), or patients with polytrauma (two or more body regions with AIS 3 or more). (Table 2, Fig. 1).

**Table 2:** Factors affecting functional-outcome change between 6 and 12 months

		Univariate				Multivariable			
	Variable	OR	95% CI		*p*	OR	95% CI		*p*
Demographics	Age	0.98	(0.97,	0.99)	0	0.99	(0.98,	1)	0.171
	Male	1.5	(1.03,	2.17)	0.032	1.36	(1.02,	1.82)	0.039
Injury score	New Injury Severity Score	0.99	(0.97,	1)	0.034	0.98	(0.96,	1)	0.136
Charlson comorbidity score	1	0.55	(0.36,	0.86)	0.008	0.65	(0.43,	1)	0.05
	2	0.37	(0.18,	0.74)	0.005	0.49	(0.21,	1.13)	0.094
	> 2	0.31	(0.12,	0.81)	0.017	0.38	(0.1,	1.42)	0.148
Pre-injury employment status	Employed	1.65	(1.17,	2.32)	0.005				
Injury mechanism	Pedestrian	0.99	(0.36,	2.71)	0.987				
	Motorcycle	1.45	(0.86,	2.42)	0.16				
	Motor vehicle	3.52	(1.48,	8.39)	0.004	2.78	(1.51,	5.12)	0.001
	Low fall	0.59	(0.42,	0.83)	0.003				
	High fall	1.09	(0.69,	1.72)	0.722				
Injury Region	Polytrauma (anatomical)	1.49	(1.02,	2.19)	0.041				
	Head and neck	0.64	(0.42,	0.97)	0.034				
	Chest	1.82	(1.23,	2.69)	0.003

Older or higher comorbidity patients had worse functional outcomes. Severity scoring on NISS, head and neck injuries (AIS 3 or more), and injuries arising from low falls were predictors of worse function.

Injury scores ISS and RTS, other injury patterns or mechanisms were not significantly associated with functional outcomes.

### Multivariable analysis

Male gender and injuries caused by motor vehicles were predictors of better outcome. All other variables (age, comorbidity, NISS), despite being significant on univariate analysis, were not significant on multivariable analysis (Table 2, Fig. 1).

### Responder and non-responder comparison

Our response rate was 31.2% (*n* = 941), with 2072 non-responders to registry officials’ telephone calls (Table 3). Non-responders tended to be foreign nationals and were younger than responders. Comorbidity scores tended to be lower for non-responders, although injury scores were similar. More non-responders suffered from polytrauma.

**Table 3:** Responder and non-responder comparison

		Non-responder		Responder		
		Number (%)/median (IQR)/mean (SD)		Number (%)/median (IQR)/mean (SD)		*p* value
Total		2072		941		
Demographics	Age	54.1	(22.5)	59.2	(20.7)	< 0.001
	Males	1467	(71%)	671	(0.71%)	0.777
Injury scores	Injury Severity Score (ISS)	21	(17, 26)	21	(17, 26)	0.135
	New Injury Severity Score (NISS)	27	(21, 34)	27	(22, 34)	0.056
	Revised Trauma Score (RTS)	7.84	(2.63, 7.84)	7.84	(3.80, 7.84)	0.006
Charlson comorbidity score	0	1595	(77%)	692	(74%)	0.017
	1	331	(16%)	159	(17%)	
	2	104	(5%)	62	(7%)	
	> 2	42	(2%)	28	(3%)	
Citizenship	Singapore citizen/permanent resident	1536	(74%)	808	(86%)	< 0.001
Mechanism of injury	Low fall	799	(39%)	443	(47%)	< 0.001
	High fall	307	(15%)	119	(13%)	0.113
	Pedestrian	78	(4%)	31	(3%)	0.522
	Cyclist	0		31	(3%)	
	Motorcycle	424	(20%)	151	(16%)	0.004
	Motor vehicle	112	(5%)	46	(5%)	0.555
	Road traffic injury	0		228	(24%)	
Injury region	Head and neck	1512	(73%)	722	(77%)	0.029
	Face	38	(2%)	20	(2%)	0.59
	Chest	595	(29%)	231	(25%)	0.018
	Abdomen and pelvic contents	274	(13%)	90	(10%)	0.004
	Extremities and pelvic girdle	375	(18%)	152	(16%)	0.193
	External	3	(0.1%)	0		–
	Polytrauma	615	(30%)	234	(25%)	0.007

## Discussion

Our study is the first study of factors associated with functional outcomes in patients with severe blunt trauma injuries in Singapore. Overall improvement rates from 6 to 12 months in our study (36%) were higher than similar studies using trauma registry data in Australia (26%) [13]. Comparison with other studies was difficult due to differences in instruments used to measure functional outcome.

Similar to international literature, we found that male gender was associated with improved functional outcomes. This approach was supported by the literature, where the female gender has been identified with worse outcomes [13, 14, 18, 25]. However, this is potentially confounded by age-related injury epidemiology. Majority of male trauma survivors were young and were involved in high-velocity injury but had good outcome. Whereas majority of female injury was associated with age-related falls, thus having worse outcome [33, 43, 44].

Although not statistically significant, patients with older age and higher comorbidity were more likely to experience deterioration in function by 12 months [12–15, 17–19, 25, 45]. This may be related to reduced physiological reserve and frail pre-injury status, thereby negatively affecting recovery. The decline in function in older patients and those with significant comorbidities could be due to natural progression of chronic disease, in addition to the impact of the injury.

Intuitively, more severe injuries would lead to worse outcomes. However, we found that trauma survivors with higher NISS, a measure of injury severity, improved better. This is likely to arise from age-related confounder as previously mentioned, and it is supported by previous literature, where long-term outcome (mortality) was not affected by NISS.

We found pre-injury employment status to be a significant predictor of improved functional outcomes. We used employment as a proxy for pre-incident functioning and socio-economic status, both of which have been previously found to be positive prognostic factors for functional improvement [6, 12, 14, 18, 20]. However, this is also potentially affected by age.

We found similar relationship patterns between anatomical injuries and functional outcomes [18]. Patients with head and neck injuries had worse outcomes, ostensibly due to concomitant spinal cord or central nervous system injury. This highlights the importance of preventive or protective strategies. Polytrauma patients were also found to experience functional improvement over time, possibly representing patients with high-velocity polytrauma requiring longer recovery time but with good rehabilitation potential. This may also explain our findings in relation to NISS and gender.

Our study also described the real-world functional outcome registry experience if majority of staff force made the calls during office hours. Our response rate at 12 months was lower than that of other countries (US 42%, UK 43%, Hong Kong 59%, and Australia 85.8%) [28, 30, 45, 46]. Younger patients and foreign nationals were more likely to be non-responders [28]. We postulate that younger patients were more likely to have recovered and returned to daily activity, hence not responding to the calls [16]. Foreign nationals may have departed the country, thereby also not responding. Conversely, patients with low falls were more likely to respond. This may be due to the fact that low falls occur more commonly in older patients who are more likely to be available during office hours or who have companions with them to answer calls. This is similar to what the Victoria State Trauma registry experienced, where the older or institutional patients tended to be captured during office hours interviews, while younger and working adults tended to be non-responders during office hours as they were working and therefore unavailable [34].

Our study was limited to trauma registry data, representing only hospitalised patients. However, as our focus was on severe injuries, we expect the majority if not all such cases to require inpatient care. Our study was able to cover all public-sector institutions nationwide, accounting for 80% of all hospital-based care in an urban multi-ethnic population. We also had issues of responder bias, where patients lacked GOSE scores at later timepoints. Nevertheless, we were able to demonstrate target groups for intervention in order to improve functional outcomes and recovery trajectories. Future studies should focus on longer-term follow-up as recovery processes may extend beyond 12 months, and on interventional strategies, such as our recent study showing that patients discharged to inpatient rehabilitation services were less likely to be readmitted [40].

## Conclusion

Females experience worse functional outcomes at 12 months, potentially due to majority of female injuries being low falls in the elderly. In contrast, motor vehicle injury patients had better functional outcomes at 12 months. Additional interventional strategies for high-risk groups should be explored [40].

## Abbreviations

AIS: Abbreviated Injury Scale
CCI: Charlson comorbidity index
GOSE: Glasgow outcome scaled-extended
ISS: Injury Severity Score
NISS: New Injury Severity Score
RTS: Revised Trauma Score
